# Salicylates in Rheumatic Affections

**Published:** 1909-03-13

**Authors:** 


### Diseases of Children.

### SALICYLATES IN RHEUMATIC AFFECTIONS.

Rheumatism in children is a frequent, virulent, and dangerous affection, usually of the heart, rarely of the joints. Chorea is very often of similar causation—according to some authorities invariably so. Scientific treatment is consequently of the utmost importance, and, until comparatively recently, we have had little more than clinical observation on which to base an estimate of the beneficial effects of drugs. Two papers read before the therapeutical and pharmacological section of the Royal Society of Medicine last December are of great interest and deserving of comment. Dr. Ralph Stockman advanced precise and definite evidence of the action of salicylates and allied bodies in the treatment of acute rheumatism. On clinical and experimental grounds he recommended large doses, because salicylic acid is rapidly excreted and is converted in the body into inert salicyluric acid. Its main poisonous action is exerted on the nervous and respiratory systems: the circulatory system is very slightly affected.

Phenol ($C_6H_5.OH$) has no action on rheumatic fever, whereas benzoic acid ($C_6H_5.COOH$), a direct derivative, has a very marked effect, though inferior to salicylic acid, which is ortho-oxybenzoic acid ($C_6H_5.COOH.OH$). Cresotinic acid ($C_6H_3.COOH.CH_3$) is also active, and so are drugs which are converted into salicylic acid in the body—namely, salicin, saligenin, acetyl-salicylic acid (aspirin), methyl-salicylate, etc. On the other hand, meta- and para-oxybenzoic acids are practically inert, although they are of the same chemical composition as salicylic acid, differing but in the position occupied by the hydroxyl group in the molecule. Other drugs, allied in character, are inert because salicylic acid is not formed from them in the tissues—namely, benzoyl-salicin (populin), methyl- and dimethyl-salicylic acids.

Dr. D. B. Lees read a paper in which the clinical and personal factors are peculiarly prominent. He entitled it "The Effective Treatment of Acute and Subacute Rheumatism," and began by stating that the treatment "as advised in text-books, and as usually carried out in practice, is inadequate and unsatisfactory." Such a sweeping assertion requires considerable justification. Probably it is based on ignorance of the methods of treatment adopted by other physicians and the limited views of individual practice. The writer recalls that over twenty years ago one of the physicians at St. Bartholomew's Hospital invariably gave his patients with rheumatic fever 15-grain doses of salicylate of soda every two hours, a dose which even Dr. Lees appears to think adequate. Considering the size and importance of this medical school, it is certain that this line of treatment, if successful, must have been adopted by many medical practitioners.

Two important points Dr. Lees insists on. The salicylate of sodium is not a depressant salt, and its reputation in that way depends on the impurities present in the earlier synthetical preparations or on the myocarditis so often present. Myocarditis is almost always present to a variable extent, more especially in children. He recommends doses of 5 to 10 grains every two hours from 6 a.m. to 10 p.m. and once during the night—that is ten doses in 24 hours—and a daily increase in each dose of 2 to 5 grains, up to a total of 20-40 grains, and occasionally more. He has given daily as much as 600 grains, combined with 1,200 grains of bicarbonate of soda, for two days to a boy of 15 years without ill-effect. In all cases each dose is combined with a double quantity of bicarbonate of soda. It can be given in peppermint or chloroform-water. The solubility of the bicarbonate is only 1 in 16, or 30 grains to the ounce of water. Larger doses must be given in larger quantities of solution, otherwise there is a risk of some doses of the alkali being inadequate. The drug must be continued for some time after the temperature has fallen, and as long as there is an evening rise.

The signs of an overdose are vomiting, drowsiness, acetonuria, acetone odour of the breath, slowing and deepening of respiration, and later, air-hunger and coma, which may prove fatal. Evil results are particularly apt to arise if the urine is too acid and if the child is constipated. The dose should not be increased unless the bowels have acted on the day the increase is ordered, and the amount of bicarbonate must be enough to render the urine alkaline. If vomiting occurs, the drug must be omitted for a few doses or as long as 12 hours.

It is extremely doubtful whether such huge doses are necessary or beneficial. Certainly the average case gets better with much less of the drug. There is no evidence that these large doses are fully absorbed, and the evil effects of constipation are a strong argument that they are not. The rapid elimination of the drug at all ages, especially in childhood, is a safeguard against over-dosage, and it is probable that as long as sufficient is given to maintain the reaction characteristic of the drug in the urine further additions are superfluous. The tests for acetonuria must be applied, if there is a possibility of symptoms arising in the course of the disease being due to over-dosage, for the acetone odour of the breath is unreliable.

There is evidence that the drug, even in large doses, will not prevent myocarditis. Dr. Lees states that the rheumatic toxæmia itself always causes more or less cardiac weakening, and that dilatation of the left ventricle is constant. These papers are valuable as indicating why certain drugs are anti-rheumatic, while allied ones have no such effect; and that under suitable precautions salicylates can be given in large doses. One would like evidence of the value of the citrate of soda, in preference to the bicarbonate, for it much more readily renders the urine alkaline. Aspirin appears to have a more prolonged effect than salicylates, because it is much more slowly excreted. It cannot be combined with an alkali. In all cases it is advisable to use alkalies with a sodium base, for potassium has a depressant action on the heart.

